# An explanatory model of factors enabling sustainability of let’s talk in an adult mental health service: a participatory case study

**DOI:** 10.1186/s13033-020-00380-9

**Published:** 2020-07-09

**Authors:** Becca Allchin, Brendan O’Hanlon, Bente M. Weimand, Fran Boyer, Georgia Cripps, Lisa Gill, Brooke Paisley, Sian Pietsch, Brad Wynne, Melinda Goodyear

**Affiliations:** 1 School of Rural Health, Faculty of Medicine, Nursing and Health Sciences, Monash University, Clayton, VIC Australia; 2 Eastern Health Mental Health Program, Box Hill, VIC Australia; 3 The Bouverie Centre, La Trobe University, Melbourne, VIC Australia; 4 Division Mental Health Services, Akershus University Hospital, Lørenskog, Norway; 5 Department of Nursing and Health Promotion, Faculty of Health Sciences, OsloMet-Oslo Metropolitan University, Oslo, Norway; 6 Emerging Minds, Hilton, SA Australia

**Keywords:** Sustainability, Let’s Talk, Case study, Adult Mental Health, Participatory research

## Abstract

**Background:**

While effective interventions have been developed to support families where a parent has a mental illness in Adult Mental Health Services, embedding and sustaining them is challenging resulting in families not having access to support. This study developed an explanatory model of influencers that had enabled sustainability of the Let’s Talk intervention in one service.

**Methods:**

A participatory case study was used to build an explanatory model of sustainability at the service using theoretical frameworks. Qualitative and quantitative data was collected about practitioner’s practice and the organisation’s implementation process and capacity to support practice. A local research group worked with the researcher using a transforming data approach through description, analysis and interpretation.

**Results:**

Influencers were grouped into four major categories: (1) *External social, political and financial context,* (2) *Resources,* (3) *Prior organisational capacity* and (4) *Sustainability Factors.* The last category, *Sustainability factors,* was divided into three subcategories: *(4.1)Practitioner* (*4.2) Organisation* and (*4.3) Parent*-*Client*. These categories form part of an explanatory model for the key influencers of continued practitioner practice and organisational capacity to support practice.

**Conclusions and implications for practice:**

In this case study, the pre-existing organisational context along with practitioner, organisation and parent-client factors operated together to influence sustainability. The results suggest that sustainability is more likely to be supported by both linking Let’s Talk to existing organisational identity, capacity, structures and relationships and by supporting mutual adaptations to improve the fit. Additionally, by understanding that setbacks are common and ongoing adjustments are needed, implementers are able to have realistic expectations of sustainability.

## Background

Research in the past two decades highlights how families are faced with greater challenges in their day to day lives when a parent experiences mental illness [1, 2]. The symptoms and treatment can disrupt a parent’s ability to attend to their children’s needs, disturbing the parent–child relationship required for healthy child development [2, 3]. Changes in roles and responsibilities in families can additionally complicate family dynamics [4]. The consequential intergenerational mental health challenges in families, including poorer outcomes for children, has led to a call for mental healthcare practices to take family-oriented perspectives [4–6]. As a result, a wide range of effective interventions tailored to different needs, population and settings has been developed [7, 8].

Let’s Talk about Children (Let’s Talk) was developed as part of a public health initiative for adult psychiatric services in Finland. It is a series of conversations between the practitioner and parent that bring into focus the wellbeing of their children while supporting the parent’s role in enabling everyday family life in the context of adversity [9–11]. Studies of Let’s Talk have focused primarily on the safety and feasibility of its use [12, 13], outcomes for children and parents [14, 15] and changes to practitioner’s practice after training [16–18]. Let’s Talk has been adapted for Australian use with freely available online training and resources (emergingminds.com.au). It was piloted and used with supported implementation in a randomised control trial (RCT) in Victoria, Australia [19, 20].

Adult Mental Health Services (AMHS) in Victoria, Australia, provide specialist clinical care for people with severe mental illness or disorders through a range of service models. One-third to one-fifth of people receiving services from AMHS are estimated to be parents of dependent children [5, 21, 22]. While parents are a significant percentage of AMHS service recipients, interventions for families where a parent has a mental illness, such as Let’s Talk, are not yet part of their regular service delivery. Organisational and practitioner factors have been identified as contributing to this gap. The models of practice common in AMHS are driven by policy and funding that focus on the adult as an individual and work in episodes of care to manage a crisis [23]. Furthermore, gaps in practitioners’ skills, knowledge and confidence to work with families [24–26], perpetuated by a lack of regular access to parents on their caseload [27], have limited the use of these interventions in everyday practice. To mitigate these, the growing body of research into implementation [28, 29], has been applied to practices for supporting parents with a mental illness [19, 30]. Sustaining such practices, however, has had less focus. It is not known what aspects of sustainability are generic to any implementation in AMHS and which may be specific to family-focused practices such as Let’s Talk. Understanding how to embed and sustain effective interventions is important for enabling families to access the support they need as part of routine mental health practices [31].

Sustainability can be understood as an ongoing adaptation process that enables the fit of an intervention within a changing context [32]. Sustainability is a desired outcome of an implementation process so that end-users continue to receive the benefits provided by the intervention, delivered by practitioners who are continuing to use the intervention, within a system that supports practitioner’s use [33]. Studying sustainability of Let’s Talk, therefore, requires a focus on both the practitioner’s practice and the organisation’s mechanisms to support practitioners use.

Greenhalgh and Papoutsi [34] advocate that health service research needs study designs that understand organisations as complex systems with dynamic interactions that also interrelate with the implementation process. Explanatory models developed from case studies can explore how and why something has happened within the complexities of real-world contexts through retrospective storytelling that provide descriptive examples of a change process [35–40]. Participatory approaches generate real-world knowledge collectively with those involved in the practices. This process can increase the legitimacy and the applicability of that knowledge to practice [41–43]. Taking a participatory approach to developing an explanatory model engages participants in the research process of analysis and interpretation to enable the findings to be put to use within their own setting [43–45]. As the research on the sustainability of health practices is yet to be applied to Let’s Talk, a participatory case study building an explanatory model of the sustainability journey within a real-word setting can support theory development in this area [46].

## Study aims

This study aimed to develop an explanatory model of influencing factors (influencers) that enabled sustainability of Let’s Talk in an AMHS with continued Let’s Talk practice. Two research questions framed the investigation; (i) what influencers enabled continued use of Let’s Talk by practitioners and (ii) what influencers enabled the continued organisational capacity for an AMHS to support practitioner’s sustained use of Let’s Talk.

## Method

### Study context

A supported implementation pilot of Let’s Talk was undertaken in Victoria, Australia in AMHS and psychiatric rehabilitation settings during 2011–2013 [20]. Following this, Let’s Talk was trialled with implementation support as part of a four-year RCT in Victorian AMHS, non-government community mental health and family support services (2013–2017) [19, 47].

Subsequent to this, two follow up studies of the eight AMHS engaged in the RCT, explored practitioners’ application of Let’s Talk after training [27] and the organisational capacity to support Let’s Talk practice [48]. The practitioner-focused study identified four AMHS with practitioners continuing to use Let’s Talk in the preceding 12 months [27]. The organisational capacity focused study scored organisations on their capacity to support Let’s Talk practice [48].

**Fig. 1:**
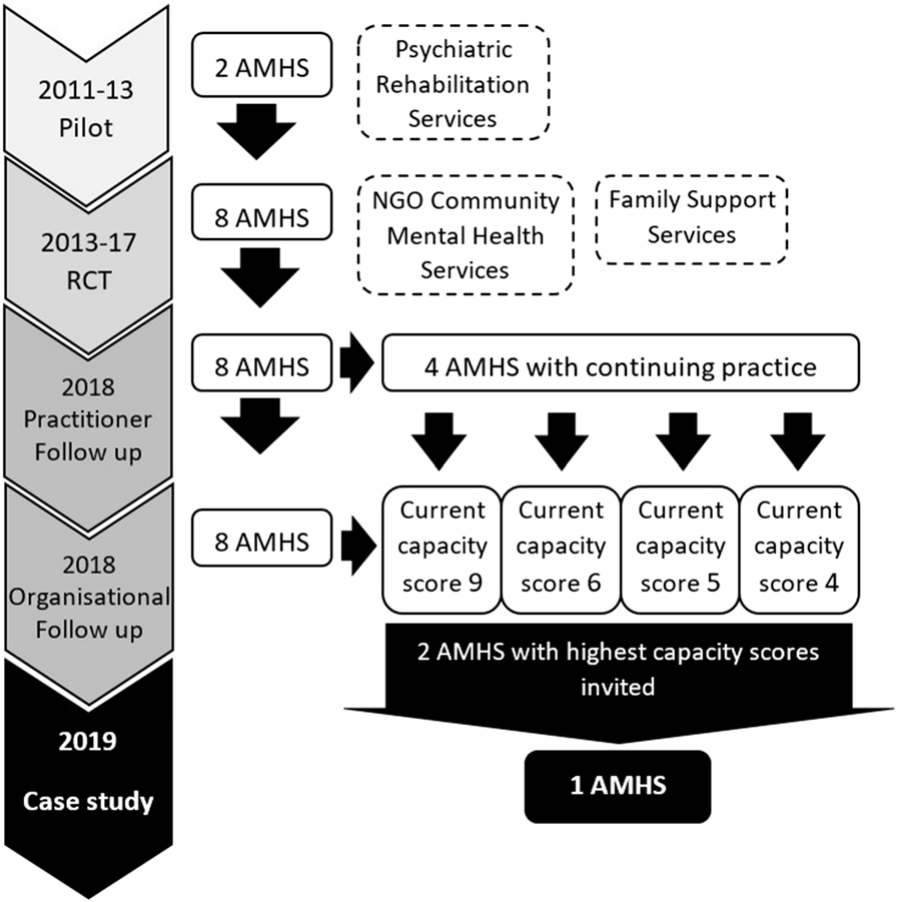
Timeline of previous studies

The current study builds on these previous studies by exploring what enabled sustainability of Let’s Talk within one of these AMHS which had practitioners continuing to use Let’s Talk and a higher capacity score (see Fig. 1).

### Theoretical approach

Using two levels of measures recommended by Scheirer and Dearing [33], this study measures sustainability as the degree to which (i) the intervention continues to be delivered in an identifiable form (albeit modified), and (ii) the organisation has capacity to support its use after its initial implementation [32, 33, 49]. Sustainability is explored as a dynamic entity (continuous adjustment of fit between the intervention and the organisation) rather than static (there or not). This view is informed by complexity thinking that understands organisations such as health care services, as dynamic, living, social systems [34, 35].

### Design

A participatory case study was used to develop an explanatory model of influencers enabling sustained practice and capacity [39, 43]. This study design was chosen as it could position the AMHS participants as co-creators of the generation of knowledge, enabling them to be active players in changing their world [39, 50, 51]. Participatory research can have a translation to practice advantage as the knowledge is co-constructed by those affected by it, thereby increasing its applicability to the practice setting [41]. AMHS have commonly been the object of, or the settings of, studies that have identified layers of barriers impeding desired change in family-focused practice as determined by others [24, 52–55]. Developing the explanatory model in partnership with the AMHS provided an opportunity for them to apply implementation science to their practice and reflect on their own sustainability journey while contributing to the production of knowledge for the scientific community [51].

The participatory approach utilised a group of key staff from within the organisation as a Local Research Group (LRG), to co-construct knowledge with the primary researcher who held a dual role as both a researcher and participant (as a service development worker within the AMHS). Selection of staff to the LRG was guided by Cargo and Mercer’s [41, p. 331] “optimal mix of partners” questions to maximise the breadth of perspectives and opportunities for translation to practice. As a result the roles identified for the LRG included: a practitioner with continued practice of Let’s Talk, a manager involved in the implementation, a FaPMI (Families where a parent has a mental illness) coordinator, Quality and Safety management personnel and a senior manager within the AMHS.

Three sustainability and implementation frameworks guided the conceptual structure of the explanatory model to provide a stronger basis for theory generation [56, 57]. These included the Consolidated Framework for Implementation Research (CFIR) [58], the Active Implementation Frameworks (AIF) [59], and the Generic Conceptual Framework for Sustainability [33].

The CFIR and the AIF provided constructs and drivers for consideration [58, 60]. The CFIR identifies key constructs compiled from implementation research across five domains; intervention/program characteristics, outer settings, inner settings, characteristics of the individuals involved and the process of implementation [58]. The AIF are designed to steer an implementation process and includes five frameworks covering the development or identification of innovations, implementation drivers, implementation stages, improvement cycles and implementing teams [61, 62].

The Generic Conceptual Framework for Sustainability that was developed by Scheirer and Dearing [33] guided the model development through providing measures and definitions of sustainability and informing how the identified influencers may have aided sustainability. Scheirer and Dearing [33] proposed that sustainability needs a more complex understanding than if the program had continued or not and detailed six conceptualisations of sustainability outcomes. These include: continuation of benefits for clients, continuation of program activities; continuation of partnerships developed, maintaining new organisational policies or practices, sustained attention to the issues and lastly program diffusion or replication [33]. Additionally, they identified three clusters of factors that affect sustainability; the characteristics of the intervention, factors in the organisational setting and factors relating to the wider environment. The Generic Conceptual Framework for Sustainability draws these key factors and potential sustainability outcomes together into hypothesised relationships. Sustainability outcomes are hypothesised as being impacted by inputs, such as the intervention and the organisation’s capacity, prior relationships and partnerships, which influence the three clusters of factors that affect sustainability.

### Sample and setting

Four of the eight AMHS engaged in the four-year RCT in Victoria, Australia [19] were identified as having practitioners with continued use of Let’s Talk [27]. The two of these four AMHS with the highest current organisational capacity score as identified in the organisational-focused follow-up study [48] were invited to participate in the case study. One of these two invited AMHS agreed to participate. See Fig. 1: Timeline of previous studies.

The study site was a large metropolitan AMHS that provides mental health assessment and interventions for people aged 25–65 years with severe mental illness in six bed-based and four community-based settings. The 75 acute beds have approximately 2190 admissions per year and 6480 clients are seen annually in the community by 300.7 effective full time (EFT) workers. Acute, continuing care, rehabilitation and specialist services are provided through a recovery model by a workforce that includes nurses, social workers, occupational therapists, psychologists, medical and lived-experience staff. Prior to any engagement of Let’s Talk, the AMHS had existing organisational structures that supported practice with family, children and carers such as an overarching policy, capacity building roles, peer support programs and a mandatory training module. The AMHS engaged in a 1-year research pilot of Let’s Talk in 2012 [20] as an opportunity to improve family-focused practice, and trial how Let’s Talk could fit within current structures. The AMHS also identified Let’s Talk as a tool that could assist the organisation move towards recovery-oriented care.

At the end of the pilot, senior management committed to participate in the four-year RCT of Let’s Talk [19] and began a series of changes to embed Let’s Talk within the model of care across the community and rehabilitation teams. These measures included: developing a Let’s Talk implementation committee and plan, creating a practice guideline directing Let’s Talk practice, adapting clinical forms and procedures, establishing Let’s Talk data collection codes and identifying a Let’s Talk training and support strategy. During the 4-year trial, the AMHS experienced significant internal changes with the introduction of the new Victorian Mental Health legislation requiring new systems, intensive retraining of staff and adaptations to everyday work. There was also significant regional growth resulting in more teams, staff changing positions and alterations to key management roles. These factors took focus away from implementation of Let’s Talk resulting in a re-implementation strategy in 2015. Another renewal occurred in 2017 with the review of the practice guideline after more shifts in key personnel had resulted in the interruption of implementation oversight.

During the RCT (2014–2015), the AMHS ran eight Let’s Talk training sessions for 107 practitioners and managers in the rehabilitation and community treatment teams. Data extracted from the follow-up study of practitioners in the eight AMHS engaged in the RCT [27], found the AMHS had 14 practitioners (seven Social Workers, three Occupational Therapists, three Nurses and one Psychologist) identified as partially delivering or completing Let’s Talk, with seven delivering Let’s Talk in the previous 12 months. Eight out of the 14 also had previous training relevant to working with families and parents including two with formal postgraduate degrees.

Training and support continue to be offered at the AMHS with seven Let’s Talk training sessions run in 2017–2018 for 54 practitioners. Seven out of nine teams had practitioners currently using Let’s Talk at the time of the study, as obtained from the AMHS three-monthly Let’s Talk data records. Let’s Talk was offered to 39 parents by 28 practitioners, with 16 practitioners starting or completing Let’s Talk with 18 parents in that time. Of the 28 practitioners who offered Let’s Talk, eight practitioners offered it to more than one parent. Half of those practitioners who offered it to multiple parents had mixed outcomes from the offer; refusal, started Let’s Talk and/or completion of Let’s Talk. There were also ten practitioners where all offers of Let’s Talk were declined.

### Procedure

The senior manager and FaPMI coordinator at the case study site identified and invited eight staff to participate in the LRG based on the criteria in the design above. Two of the practitioners invited were unable to be released, leaving a group of six plus the researcher. Prior to attending the first meeting, the researcher engaged the members via email and sent a companion guide outlining definitions, the study context, method and known data and guidelines for group engagement to enable reflexivity. Attendance of the LRG at each session varied from four to six, as detailed in Additional file 1: 1.1 Local Research Group Participants & Attendance. Where possible the primary researcher engaged absent participants between sessions.

Five sessions were coordinated over an 11 week period between Feb and April 2019. The face to face sessions ran for 1.5–2 h per session at the case study site. Each session included a warm-up activity to help with group cohesion, a reflection time on the knowledge generated previously and data collected in-between, as well as an activity to meet the session objective. The first two sessions focused on data generation through developing a shared understanding of the implementation process (session 1) and developing a shared understanding of influencers of continued practice and capacity (session 2). The last three sessions focused on analysis and interpretation through comparing the identified key influencers to literature (session 3), prioritising key influencers (session 4) and refining the explanatory model and future planning (session 5). A detailed session plan for each meeting was developed outlining the objective, core tasks, activities and measures and is attached as an Additional file 1: 1.2 Case Study Session Outline. However an example of session two and three can be seen in Table 1.

**Table 1:** Case study outline: session example

Session no.	Session objective	Tasks	Activities	Measure of aim met	Measure of participation process
2 (1.5 h)	Develop a shared understanding of influencers of continued practice and capacity	Re-establish team cohesion	Warm-up by FaPMI coordinator		
		Review collected data Practitioner use Implementation journey Implementation documents	Review practitioner data—phase 2 and current data Review assumptions and ideas raised in implementation journey activity Create space for further questions to be raised	More questions raised	Analysis of sticky notes Framelaps and audio End of session feedback form
		Introduce the Generic conceptual framework for sustainability used for coding	Gave a rationale for using General conceptual framework for sustainability for coding data from implementation journey activity and presented developing theme matrix		
		Identify influencers of continued practice	Each brainstorm with own sticky notes (initial at bottom). Different colour for practice and capacity Place on the wall so can be seen by all Cluster influencers into categories using group consensus	Matrix of influencers	
		Identify influencers of continued capacity			
3 (1.5 h)	Compare key influencers to literature	Re-establish team cohesion	Warm-up	Refined theme matrix with descriptions matching data	Framelaps and audio Work done in each pair End of session feedback form
		Present established frameworks that have shaped the theme matrix	Present the CFIR, Active Implementation Frameworks and refresh re General conceptual framework for sustainability		
		Review and refine developing themes matrix	Pairs reviewed a section of themes, their description and data to check: Themes reflected the data If the themes picked up on what they had wanted to convey about key influencers Decide if theme should be kept, rolled into another or removed		

The researcher with the LRG followed Wolcott’s [63] approach of transforming data through description, analysis and interpretation. These three processes, which are neither discrete nor necessarily sequential [43], were addressed in this study through a series of four cyclical phases; (1) developing a timeline of implementation, (2) document collecting, (3) concept mapping and (4) explanation building [43].

1. Develop a timeline of implementation: Firstly a collective understanding of the process of implementation at that site was developed. Using the River of life tool [64], the LRG with the researcher pictorially documented the implementation journey highlighting key influencers and identifying gaps in the knowledge that needed further data (i.e. identifying practitioners continuing to use Let’s Talk) (Session 1–2). During this phase, the LRG identified documents that could give further information about that journey such as implementation plans, communication memos and snapshot data about current use of Let’s Talk.
2. Document collection: The researcher collected identified documents and used the questions raised in the previous session to review and summarise the content. Summarised data was tabled at the next session. (Session 1–3). Reflective notes and memos were made by the researcher after each session and in relation to data collected (Session 1–5).
3. Concept mapping: The LRG with the researcher compared the generated and collected data against the three identified implementation and sustainability frameworks to refine the influencers and map interrelationships and patterns (Session 2–4).
4. Explanation building: The LRG with the researcher built the influencers, patterns and interrelationships into an explanatory model and explored its meaning to them as individuals and for the service (Session 3–5).

### Data collection

Case studies collect data from multiple sources with flexibility to make adjustments as the process develops, enabling depth and triangulation of evidence [39]. Beginning with data gathered from the two follow up studies of AMHS services [27, 48], this case study also used evidence from three other sources: (i) data generated in meetings including a timeline of implementation, collectively agreed on influences as well as audio and photographic records of each session (ii) the primary researcher’s reflective notes and summary memos from meetings and (iii) organisational documents including implementation plans, policy, service memos, snapshot audits of practice and training plans (see Table 2).

**Table 2:** Table of documents

Document	Purpose
2012 Practice guideline Let’s Talk about Children	Guide for regular clinical practice with service targets of use and monitoring
2013 Let’s Talk and RCT implementation process 2012–2013	To oversee research and establishment process
2013 Let’s Talk implementation process action plan Oct 2013	
2013 Let’s Talk implementation process timeline	
2014–2015 Let’s Talk training records	Recording training sessions and attendees
2015 05 Let’s Talk Memo	Communication with staff about activities of RCT research and implementation
2015 06 Let’s Talk Memo	
2015 07 Let’s Talk Memo	
2016 05 Let’s Talk sustainability Excel sheet	Let’s Talk use monitoring tool
2017 Let’s Talk implementation tasks 2017	Re-establish implementation process
2017 03 Briefing paper AMHS Let’s Talk sustainment plan	Re-establish implementation process
2017 04 Let’s Talk memo sent	Communication with staff about expectations of and support for practice
2017 Let’s Talk implementation timeline 2017	Re-establish implementation process
2018 04 Let’s Talk Memo	Communication with staff about expectations of, and support for practice
2018 11 Let’s Talk Memo	
2019 01 Let’s Talk Memo	

Audio-recording were not transcribed but were used directly, as inspired by Halcomb and Davidson [65], to check for consistency of emerging explanations with participants’ descriptions. All data were entered into NVivo qualitative data analysis software (QSR International Pty Ltd. Version 12, 2018) as it can support diverse data collation and direct data management.

### Data analysis

Thematic analysis, a theoretically flexible approach [66], was used to identify patterns across all data. Analysis began during data collection, as the emerging explanatory story was compared in an iterative process against constructs from frameworks of sustainability [33, 39, 43, 58, 61]. In participatory case study analysis, the process of participation determines what the data is and how it fits with the theory and frameworks. As a result model development process is interwoven with the final results. The analysis process included a back and forth pattern of group activities generating and analysing data and the researcher reviewing and analysing data between meetings. Each meeting began with the researcher presenting data and analysis back to the group for discussion to support rigor and reflexivity.

The researcher created an initial framework after inductively coding data from session one. This data included the pictorial description of the timeline and process of implementation of Let’s Talk along with the initial identification of key influences as developed by the LRG. The session’s audio was used for clarification. Coding was facilitated through the use of NVivo. The key influencers that were generated by the LRG in session two, were thematically coded by the researcher resulting in adaptation of the emerging framework. In subsequent meetings, the codes and framework were reviewed in the light of frameworks of sustainability [33, 58, 61] and refined by the researcher together with LRG to categorise and define the influencers and develop an explanatory model. The final model was reviewed by all researchers and agreed upon in its entirety.

## Results

The process and method of the study, as previously described, form part of the findings of the case study. The influencers and the explanatory model will be presented next, including relationships identified between the influencers. Direct quotes from the participants were not collected as part of the data but where possible examples from the raw data generated by participants will be included to illustrate how the model was developed. The two research questions resulted in a common collection of influencers which were developed into the explanatory model.

### Identification of influencers

The finalised list of influencers agreed to by the LRG were organised under four major categories informed by the Generic Conceptual Framework for Sustainability [33]. These categories included (1) *External social, political and financial context*, (2) *Resources,* (3) *Prior organisational capacity* and lastly (4) *Sustainability Factors* which was divided into three subcategories of factors: (4.1) *Practitioner*, (4.2) *Organisation* and (4.3) *Parent*-*Client.* The first three categories of influencers, relating to the external environment, the pre-existing organisational structures and the resource context, reflected the context into which the implementation of Let’s Talk occurred. During the development of the implementation journey in session one, the LRG identified this context, as creating a fertile ground into which the implementation could take place. Within the category of *Prior organisational capacity,* for example, the organisation’s *Existing relationships and partnerships* within the field of families, children and carers were identified by the LRG as influencing the organisation’s access to resources and its openness to opportunities to engage in new innovations relating to family-focused practice. In a further illustration, the LRG identified that within the category of the *External social, political and financial context,* a window of opportunity occurred with the establishment of statewide mental health reforms. In specifying the responsibilities of AMHS to support children of parents receiving their services, the reforms provided an authorising environment to integrate interventions like Let’s Talk into practice. In another example, within the category of *Resources*, the LRG identified how access to the new online resources for Let’s Talk provided free modules of training for practitioners and free resources for parents making the intervention’s implementation more affordable.

The three subcategories of *Sustainability factors* reflected how the practitioners, the organisation itself and parent-clients influenced the implementation and sustaining of Let’s Talk. For example, the LRG described how practitioners using Let’s Talk had acted as role models for other practitioners and normalised the work as part of standard practice. Accountability structures within the organisation were described by the LRG as communicating priority and generating data that was used to drive practice change. The LRG identified that when parent-clients specifically requested assistance with parenting and children, practitioners were prompted to use the practice (see Table 3).

**Table 3:** Descriptions of enabling influencers of continued organisational capacity and practitioner use

Category	Enabling influencer	Description
External social, political, financial context	External social, political, financial context	A new political and policy direction (new MH Act, Recovery frameworks, increased MH funding), new national workforce initiative (COPMI (Children of Parents with a Mental Illness) online resource development) and a new research agenda (Government funded RCT on recovery and parenting) were external context enablers for the organisation and the intervention
Prior organisational capacity: organisation history prior to implementation	Existing organisational structures	Existing organisational structures to support family, children and carer focused work enabled the new intervention to fit. These structures included family, children and carer specific capacity-building roles within the organisation for over 10 years, as well as policy and mandatory training systems to uphold policy
	Existing relationships and partnerships (organisational bridging social capital)	Influential relationships and partnerships enabled prior and continued organisational capacity through bridging the organisation to opportunity and innovation in the field of family, children and carers (training, research, resource development, expanded relationships with universities, government, international experts)
	Organisational ownership	Organisational ownership of implementation was enabled through the development of own implementation vision and plans and being a steering partner in the research
	Prior organisational identity	Organisational reputation and brand prior to implementation was already family, children and carer focused with a history of carer support that included children’s voices, of parent-focused work and programs for children. The organisation’s identity also included using research for learning
Resources	Resources	Funding, staffing or other resources enabled sustained practice and organisational capacity. Growth funding increased practitioner to client ratio and enabled recovery resources. Research brought funding, attention to issue and resources for data and analysis. National workforce initiative enabled accessibility through high quality, standardised online training and free resources for parents
Sustainability factors: practitioner: factors about the practitioners that enable sustainability	Parents on caseload	Practitioner’s opportunity to use Let’s Talk was influenced by having parents on their caseload. While demographics of region/team affect % of parents, practitioner’s previous experience, interests and comfort can result in self-selection of parent clients
	Models of practice used by practitioners	A person, parent and family-focused model of practice that attends to relationships enabled practitioners to incorporate parenting and recovery into their work.
	Support from peers	Other practitioners doing Let’s Talk provided role models, normalised the work, built acceptability and critical mass amongst peers and enabled practitioners to see it is possible to do within pressures of everyday work
	Practitioner characteristics	Practitioners professional interests, prior experience & training in family, children and carer work and life/personal experience influenced use
	Practitioner identity	Practitioners are enabled to use Let’s Talk when they are connected and have satisfaction in their role, identify as a good practitioner and have individual accountability for their practice
Sustainability factors: organisational: sustainability influencers related to the organisation	Accountability structures	Having organisational structures to drive accountability supported the sustainability of organisational capacity and practitioner use. Such organisational structures included a driving committee embedded into the organisational hierarchy, capacity development personnel and system embedded into the service, a policy communicating priority and core business, systems monitoring policy use, data being used as a driver of practice and compliance with policy
	Leadership accountability	An expectation of leaders to lead was supported through involvement in training, reporting and support systems and reflected in adherence to strategic directions, policy and programs
	Leadership stability	Stability in leadership allowed for organisational memory and continued commitment, while new leadership within stability brought new energy
	Organisation fitting the intervention to self	Adaptations were made by the organisation to better the fit of Let’s Talk such as integrating documentation, system prompts, policy development and data reports
	Organisational identity	Let’s Talk was aligned with the organisation’s reputation and brand which included a recovery family-oriented culture that valued lived experience and had connections, strategic partnerships and relationships that enabled learning and innovation. These were upheld by leadership and reflected in strategic directions, policy and programs
	Other organisational initiatives	There is a synergy between other initiatives active in the organisations that supported use such as peer leadership, introducing a recovery model and a focus on data documentation
	Team leadership support	All levels of leadership (including informal) supported sustained practice through buffering changes at internal/external level to manage workload, aiding workforce stability, upholding priority set by the organisation, holding practitioners to account and creating a culture that was open to practice and that can see how it could fit into current practice
	Training and practice support	The organisation had regular and accessible training that was integrated into data systems and other training. The selection of participants was purposeful and delivery methods incorporate peer facilitators. There were post-training reflective spaces and support that linked to other initiatives and gave attention to measure and build competency
Sustainability factors: parent client		The parent client’s stage of recovery and willingness to request help with parenting and children influenced uptake

### Explanatory model

Work on the explanatory model commenced after the development of the implementation timeline and initial identification of influencers in session one. The researcher and the LRG selected the Generic Conceptual Framework for Sustainability [33] to inform the model development, due to the alignment of its components and structure to the focus in session one on what occurred prior to implementing Let’s Talk. The final explanatory model describes what had enabled practitioners continued use of Let’s Talk and the organisational capacity to support its use at that AMHS.

**Fig. 2:**
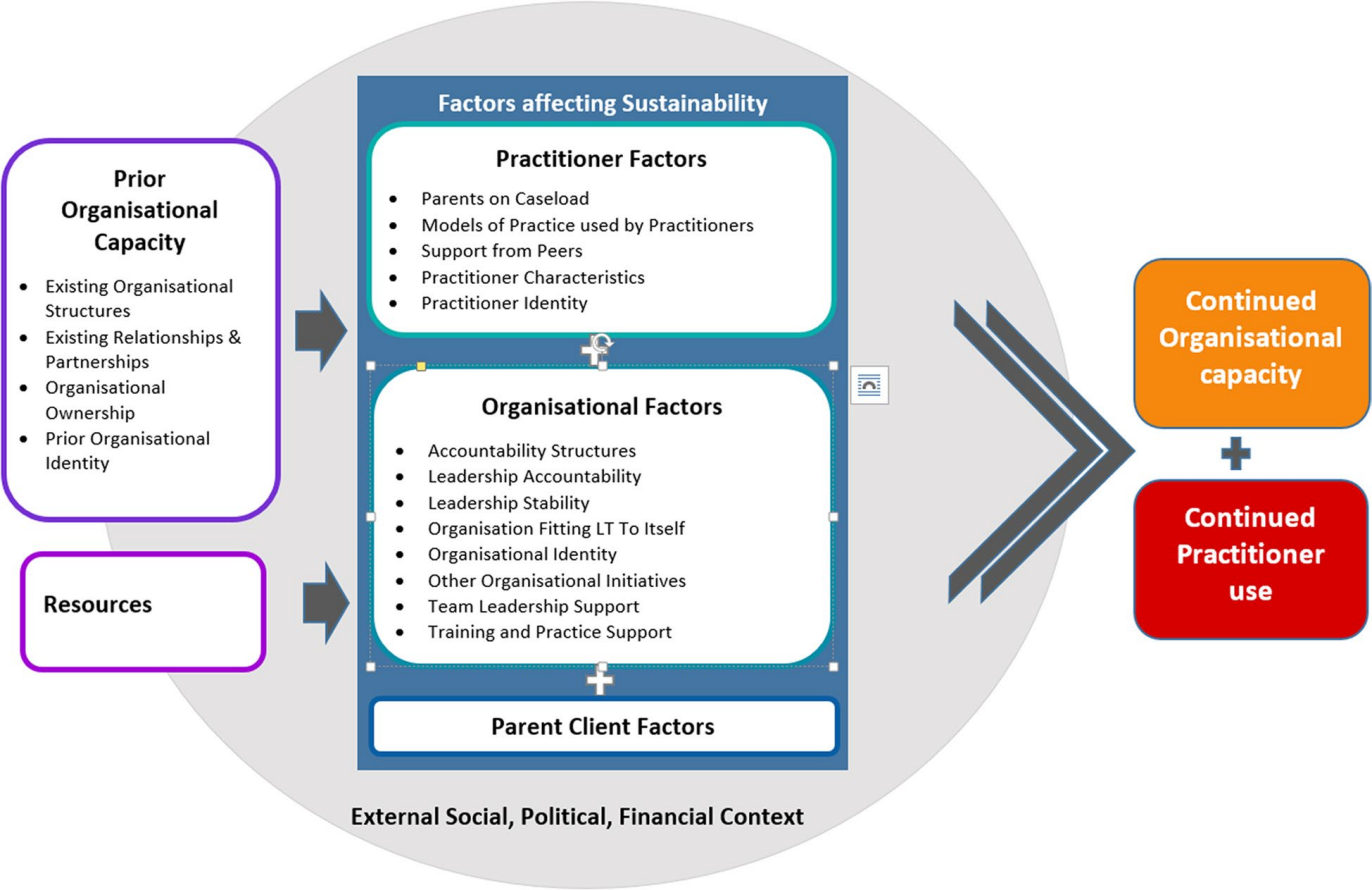
Explanatory model

The model illustrates how the existence of *Resources* and the established *Prior organisational capacity* created a foundation for *Factors affecting sustainability.* The *Prior organisational capacity* was defined by their existing structures, relationships and partnership, organisational ownership of the implementation process and prior organisational identity. The *Factors affecting sustainability,* whilst described in three categories; the *Practitioner,* the *Organisation* and the *Parent*-*Client,* were understood by the LRG to work in synergy to provide the sustained outcome. All of these influencers were described as being situated within a broader *External, social, political and financial context* in which a number of coinciding events acted as enablers (see Fig. 2).

### Relationships

While the LRG worked to define each influencer as a unique entity, a number of influencers were identified as interconnected. For example, the *Organisational identity* as a family-oriented service that values lived experience was seen through *Accountability structures* such as the Family, Children and Carers Policy and the Mental Health Program’s Consumer, Carer, Family and Children Advisory Committee which communicated prioritisation of work such as Let’s Talk. These additionally were understood by the LRG to give frameworks for *Leadership accountability* by providing expectations for leaders to enact policy and strategic directions which was supported by having reporting expectation. These relationships were defined and are represented in Table 4.

**Table 4:** Relationships between influencers

Influencer	Influencer	Relationship
Organisational identity	Leadership accountability	Organisational identity created a structure to enable leadership accountability to be upheld and leadership upheld the organisational identity
	Accountability structures	The accountability structures were expressions of the organisational identity (i.e. policy communicating priority and core business)
	Practitioner identity	The idea that the individuals in an organisation shape the organisational identity and culture and yet the organisational identity/branding attracts certain sort of people. Need a certain amount of individuals who value and do family-focused practice for the organisation to continue to represent their projected identity
	Team leadership support	Organisational identity shaped the leadership opportunities and way leaders led
	Existing relationships and partnerships	Having relationships and partnerships with universities built an identity of a learning culture, brought in new ideas and helped the organisation have a brand of learning culture. Existing relationships and partnerships have a continuous role in the organisation’s identity
Practitioner identity	Existing relationships and partnerships	Practitioner identity shaped the relationships and partnerships the organisation had, while at the same time the organisation’s relationships and partnerships provided opportunities for workers to expand and grow in their identity
	Parents on Caseload	Practitioner’s interests, sense of who they are and how they practice influenced the sort of clients they are allocated
Parents on caseload	Team leadership support	Leadership had mechanisms to shape caseload and enable practitioners to have parents on their caseload
Accountability structures	Leadership accountability	Accountability structures are mechanisms for accountability while Leadership accountability relates to people. Leaders held to account helped to uphold the accountability structures and the structures enabled leaders to be able to be held to account
Resources	External social, political and financial context	Increase in focus on mental health in state government lead to growth funding across the state giving the service more funding and enabling changes to practice (more staffing/new positions/new models)

### Prioritisation of influencers

**Fig. 3:**
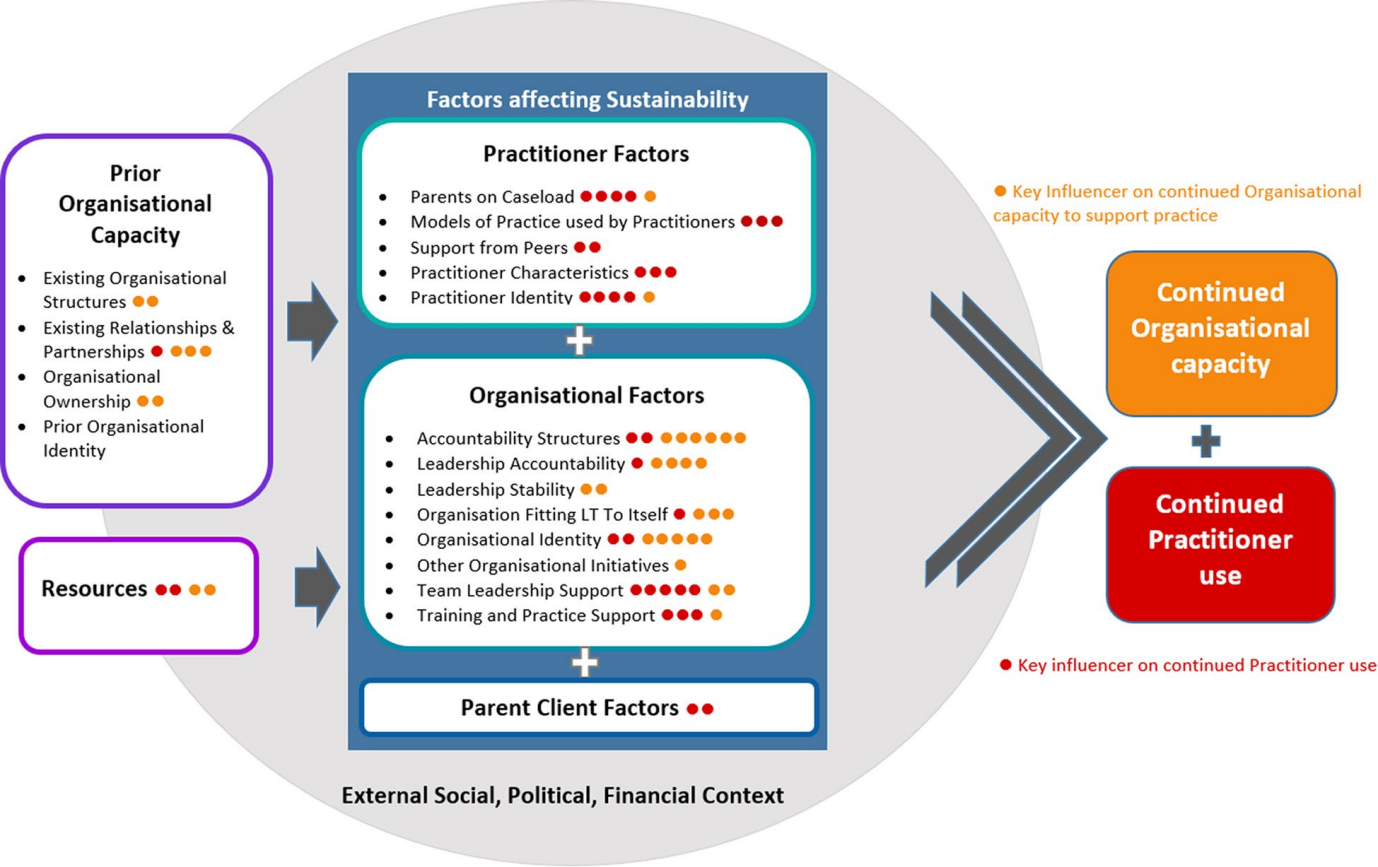
Explanatory model with prioritised influencers

Each member of the LRG was asked to identify five key influencers impacting practitioner use and five key influencers impacting organisational capacity. These were plotted against the explanatory model as a way of highlighting differences and agreement of group members as well as exploring the influencer’s impact on practice as opposed to organisational capacity (see Fig. 3).

As seen in Fig. 3, practitioner use was understood to be impacted by both *Practitioner* and *Organisational* sustainability factors while organisational capacity was impacted primarily by *Organisational* sustainability factors and the *Prior organisational capacity*.

## Discussion

This study developed an explanatory model of what enabled sustainability of Let’s Talk in one AMHS through exploring influencers that enabled (i) practitioner’s continued use of Let’s Talk and (ii) the organisation’s capacity to support continued practitioner use. The model was developed through a participatory process in partnership with people in the setting in which the model can be applied. In this way, the model generation process is as important as the final product, as it is developed to enhance sustainability in real-world AMHS settings in ways identified by those who work with them.

While specific to Let’s Talk, the explanatory model has implications for any innovation in AMHS settings. The explanatory model particularly highlights how the organisation’s history contributed to enabling sustainability. Alignment between the organisation and an innovation is known to increase the likelihood of sustainability [37] and in this setting, Let’s Talk was seen as a continuation of previous practice with family, children and carers. The organisation’s pre-existing influential relationships and partnerships in the field of family, children and carers, enabled organisational openness to a new innovation and access to resources for implementation of Let’s Talk. This suggests that Let’s Talk is more likely to be sustained when linked to an organisation’s pre-existing identity, capacity, structures and relationships.

The complex and multifactorial nature of sustainability influencers displayed in the model is consistent with the sustainability and implementation literature [37, 58, 61, 67]. At this AMHS, sustained practitioner practice was understood as being particularly influenced by both practitioner and organisational factors. *Practitioner identity*, *Characteristics* and *Existing models of practice* (i.e. family-centred approaches) shaped their interest, influenced who was on their caseload and affected their likelihood to deliver Let’s Talk. *Training and practice support* built competence and confidence, while *Team leadership* reinforced service priorities, created expectations and communicated how Let’s Talk fitted into everyday practice. Leadership’s role in understanding interventions and problem solving to support integration into everyday practice has been acknowledged as pivotal for successful implementation and sustaining practice change [68–72].

Continued capacity to support practitioners’ use of Let’s Talk was understood in this AMHS as being mainly influenced by a combination of organisational factors such as *Organisational identity, Accountability structures* and *Leadership accountability*. Having an identity as a recovery and family-oriented organisation provided a context for making Let’s Talk a priority. The accountability structures further communicated that priority through committees, policy and reporting systems. This created expectations and built accountability systems for leadership. These reflect leadership’s role in communicating priorities and establishing an organisational culture open to change [68, 73]. Changes made to documentation, policy and systems to fit the intervention to the AMHS, represent the mutual adjustment of the innovation to the setting and the setting to the innovation, identified as a key for sustainability [33, 49].

While leadership is a critical director of organisational culture [69, 74], the explanatory model explored the influence individuals can have in shaping the organisation and its culture. Organisational culture and identity attracts individuals with certain models of practice and practitioner identity. At the same time, however, organisations are made up of individuals that bring their unique skills and connections that can shape the direction of an organisation. In this AMHS, experienced family-focused practitioners contributed to the organisational culture and identity through influencing policy development, introducing new service delivery models and linking the organisation to innovation through research partnerships. Practitioner’s identity and existing practice models are not easily changed by training and practice support, so staff selection is advocated to ensure practitioners have the desired characteristics [75]. Whilst an emphasis on family-focused practice is not an explicit staff selection criteria in this AMHS, the model development process illuminated how this could support future sustainability of Let’s Talk. Additionally, attention in the recruitment process to the individual’s relationships and connections can enable organisations to create a bridge to new resources and opportunities [76], strengthening sustainability.

Many of the influencers identified by the LRG are common to implementing and sustaining practice change more broadly, however, there were aspects that were specific to Let’s Talk. Most LRG members did not rate *Parent*-*client factors* as highly influencing sustainability of Let’s Talk and where it was seen, it was influencing practitioner practice. LRG reflections suggested that help-seeking for parenting and children may not be common in AMHS. Stirman et al. [37] reflect that consumers of mental health services are often unaware of evidence-based psychosocial treatments and their perspectives on implementation and sustainability are underrepresented in theory and research. While people with lived experience of mental health issues have been central to the development of recovery models and there is a growing focus on co-development in health care [77–80], these results suggest that further research on how parent-clients can influence practitioner’s Let’s Talk practice and drive organisational capacity would seem warranted. The reflective space created by the participatory process of the model development, enabled the AMHS to consider how to utilise the perspectives of people with lived experience to further sustainability.

The influencers and model highlight the intertwined nature between factors, reinforcing the need to embrace complexity [34] and explore sustainability and implementation in ways that look at the ‘whole’ system. Often sustainability efforts can be immobilised by a ‘blame game’, wherein individuals are blamed for not adopting new practices or organisational barriers are identified as limiting individuals uptake of new practices. Without the intention to understand the parts within their context, it is easy for one of those sets of factors to be positioned as ‘the reason’ an intervention is not sustained, blocking fruitful exploration and problem-solving. The case study method allowed for an ‘in depth’ exploration of the complexity of sustainability of Let’s Talk in this AMHS and highlighted the interaction of the multilevel influencers that may impact sustainability of any innovation in an AMHS setting.

The development of the explanatory model gave an opportunity to apply implementation and sustainability concepts to practice. In the process, it illuminated the nature of sustainability. While charting the timeline of implementation, it became apparent that much change had occurred. Political and policy fluctuations changed the external environment, while internally, the organisation was shaped by changes to structure and personnel at all levels. This resulted in a need for reviewing and restarting implementation plans and building understanding in all levels of leadership in order to enable team leadership support, leadership accountability and accountability structures. Understanding that the implementation of new practices happens within a constantly changing environment [32, 37, 81], allows organisations to build realistic expectations that anticipate and plan for the ongoing adjustments that are needed to fit an intervention to current practice. Recognising this is part of real-world implementation can preserve hope, help to keep momentum through disruptions and guide monitoring and accountability structures.

Using an explanatory model to explore what had enabled sustainability even in the context of multiple barriers, was seen by the LRG as an encouraging way to focus on the next steps in their sustainability journey of Let’s Talk. The process gave the organisation an opportunity to look at what had worked well, what the current status of the intervention was and what could be leveraged in the future. Consistent with other strengths-based inquiry and participatory research models [82], the research process itself built a sense of empowerment that facilitated application of research, highlighting the usefulness of research processes that build participant’s capacity.

## Strengths and limitations

This case study’s development of an explanatory model gives rich insight to mechanisms at work within one service which can inform future implementation planning in other AMHS. While applicable to other settings, the study only attempts to explain how sustainability of Let’s Talk occurred in one setting.

The LRG were selected for their own unique perspectives, rather than representing a group of people. The workplace demands resulted in varying members of the LRG being able to attend the five meetings, limiting the breadth of input to the discussion and development of the model. This was mitigated by the researcher communicating between meetings to share the process of the session and gain the absent members unique perspective.

While barriers or challenges to sustainability were also discussed, there was no attempt to balance the barriers with the enablers of sustainability. Instead, the development of an explanatory model of sustained practice focused on what had enabled the sustained practice and capacity that the organisation had within the context of these barriers.

## Conclusions

Implementing practice change is difficult in health settings with changing environments and many known barriers. Existing literature has a limited focus on what happens after implementation of family-focused interventions in AMHS and little is known about what helps to sustain practice of Let’s Talk. The explanatory model developed in this study offers a picture of what influenced sustainability in one AMHS in the context of real-world barriers. The reflective space also extended to consideration of how this model could be used to implement and sustain other clinical practices.

Historically there has been a strong focus on what gets in the way of family-focused practice and very little on what supports uptake and sustainability. As seen in this study, a positive inquiry approach that looks for enablers and strengths has the potential to build enthusiasm and momentum within the organisation, aiding the sustainability quest. Learning across multiple levels allows for different voices to be heard, bringing richer learning and more opportunities for supporting change. By focusing on what helps to sustain practice, organisations can amplify strategies that have already helped whilst working within the already known barriers to sustainability. Whilst the findings have implications for implementing and sustaining Let’s Talk, they also have broader implications for sustaining any change process in AMHS settings.

## Supplementary information

**Additional file 1.** Case Study participantion details (1.1) and session outline (1.2).

## Data Availability

The data that support the findings of this study are available from Melinda Goodyear at Melinda.goodyear@monash.edu but restrictions apply to the availability of these data, which were used under license for the current study, and so are not publicly available. Data are however available from the authors upon reasonable request and with permission of Melinda Goodyear.

## Abbreviations

AMHS: Adult Mental Health Services
CFIR: Consolidated Framework for Implementation Research
EFT: effective full time
FaPMI: Families where a Parent has a Mental Illness
Let’s Talk: Let’s Talk about Children Intervention
LRG: Local Research Group
RCT: randomised control trial
Influencers: factors influencing sustainability

## References

[1] Falkov A, Goodyear MJ, Hosman CMH, Biebel K, Skogøy BE, Kowalenko NM (2016). A systems approach to enhance global efforts to implement family-focused mental health interventions. Child Youth Serv..

[2] Reupert AE, Maybery DJ (2016). What do we know about families where parents have a mental illness? A systematic review. Child Youth Serv..

[3] van Santvoort F, Hosman CMH, Janssens JMAM, van Doesum KTM, Reupert A, Loon LMA (2015). The impact of various parental mental disorders on children’s diagnoses: a systematic review. Clin Child Fam Psychol Rev..

[4] Martinsen EH, Weimand BM, Pedersen R, Norvoll R (2019). The silent world of young next of kin in mental healthcare. Nurs Ethics..

[5] Maybery DJ, Reupert AE (2018). The number of parents who are patients attending adult psychiatric services. Curr Opin Psychiatry..

[6] Tabak I, Zabłocka-Żytka L, Ryan P, Poma SZ, Joronen K, Viganò G (2016). Needs, expectations and consequences for children growing up in a family where the parent has a mental illness. Int J Ment Health Nurs..

[7] Siegenthaler E, Munder T, Egger M (2012). Effect of preventive interventions in mentally ill parents on the mental health of the offspring: systematic review and meta-analysis. J Am Acad Child Adolesc Psychiatry.

[8] Marston N, Stavnes K, Van Loon LMA, Drost LM, Maybery DJ, Mosek A (2016). A content analysis of Intervention Key Elements and Assessments (IKEA): what’s in the black box in the interventions directed to families where a parent has a mental illness?. Child Youth Serv.

[9] Beardslee WR, Solantaus T, Morgan BS, Gladstone TR, Kowalenko NM (2012). Preventive interventions for children of parents with depression: international perspectives. Med J Aust.

[10] Solantaus T, Reupert AE, Maybery DJ, Reupert AE, Maybery DJ, Nicholson J, Gopfert M, Seeman MV (2015). Working with parents who have a psychiatric disorder. Parental psychiatric disorder: distressed parents and their families.

[11] Solantaus T, Toikka S (2006). The effective family programme: preventative services for the children of mentally ill parents in Finland. Int J Ment Health Prom.

[12] Solantaus T, Toikka S, Alasuutari M, Beardslee WR, Paavonen EJ (2009). Safety, feasibility and family experiences of preventive interventions for children and families with parental depression. Int J Ment Health Prom.

[13] Ueno R, Osada H, Solantaus T, Murakoshi A, Inoue T (2019). Safety, feasibility, fidelity, and perceived benefits of an intervention for parents with mood disorders and their children—“Let’s Talk About Children” in Japan. J Fam Psychother..

[14] Punamäki R-L, Paavonen J, Toikka S, Solantaus T (2013). Effectiveness of preventive family intervention in improving cognitive attributions among children of depressed parents: a randomized study. J Fam Psychol..

[15] Cooper V, Reupert AE (2017). “Let’s Talk About Children” resource: a parallel mixed method evaluation. Soc Work Ment Health..

[16] Tchernegovski P, Reupert AE, Maybery DJ (2015). “Let’s Talk about Children”: a pilot evaluation of an e-learning resource for mental health clinicians. Clin Psychol..

[17] Toikka S, Solantaus T (2006). The Effective Family Programme II: clinicians’ experiences of training in promotive and preventative child mental health methods. Int J Ment Health Prom..

[18] Karibi H, Arblaster K (2019). Clinician experiences of “Let’s Talk about Children” training and implementation to support families affected by parental mental illness. J Ment Health Training Educ Pract.

[19] Maybery DJ, Goodyear MJ, Reupert AE, Sheen J, Cann W, Dalziel K (2017). Developing an Australian-first recovery model for parents in Victorian mental health and family services: a study protocol for a randomised controlled trial. BMC Psychiatry..

[20] Maybery DJ, Goodyear MJ, Reupert AE, Sheen J, Cann W, O’Hanlon B (2019). A mixed method evaluation of an intervention for parents with mental illness. Clin Child Psychol Psychiatry..

[21] Maybery DJ, Nicholson J, Reupert AE, Reupert AE, Maybery DJ, Nicholson J, Gopfert M, Seeman MV (2015). Parental mental illness: estimating prevalence to inform policy and practice. Parental psychiatric disorder: distressed parents and their families.

[22] Maybery DJ, Reupert AE, Patrick K, Goodyear M, Crase L (2009). Prevalence of parental mental illness in Australian families. Psychiatr Bull.

[23] Allchin B, Goodyear M, O’Hanlon B, Weimand BM (2020). Leadership perspectives on key elements influencing implementing a family focused intervention in mental health services. J Psychiatr Ment Health Nurs..

[24] Maybery DJ, Goodyear MJ, Reupert AE, Grant A (2016). Worker, workplace or families: what influences family focused practices in adult mental health?. J Psychiatr Ment Health Nurs.

[25] Tchernegovski P, Hine R, Reupert AE, Maybery DJ (2018). Adult mental health clinicians’ perspectives of parents with a mental illness and their children: single and dual focus approaches. BMC Health Serv Res..

[26] Goodyear MJ, Maybery DJ, Reupert AE, Allchin R, Fraser C, Fernbacher S (2016). Thinking families: a study of the characteristics of the workforce that delivers family-focussed practice. Int J Ment Health Nurs..

[27] Allchin B, O’Hanlon B, Weimand BM, Goodyear MJ (2020). Practitioners’ application of Let’s Talk about Children intervention in adult mental health services. Int J Ment Health Nurs..

[28] Moullin JC, Sabater-Hernández D, Fernandez-Llimos F, Benrimoj SI (2015). A systematic review of implementation frameworks of innovations in healthcare and resulting generic implementation framework. Health Res Policy Syst..

[29] Nilsen P (2015). Making sense of implementation theories, models and frameworks. Implement Sci..

[30] Skogøy BE, Sørgaard K, Maybery D, Ruud T, Stavnes K, Kufås E (2018). Hospitals implementing changes in law to protect children of ill parents: a cross-sectional study. BMC Health Serv Res..

[31] Maybery DJ, Foster K, Goodyear MJ, Grant A, Patraporn T, Skogy BE, Reupert AE, Maybery DJ, Nicholson J, Gopfert M, Seeman MV (2015). How can we make the psychiatric workforce more family focused?. Parental psychiatric disorder: distressed parents and their families.

[32] Chambers DA, Glasgow RE, Stange KC (2013). The dynamic sustainability framework: addressing the paradox of sustainment amid ongoing change. Implement Sci..

[33] Scheirer MA, Dearing JW (2011). An agenda for research on the sustainability of public health programs. Am J Public Health.

[34] Greenhalgh T, Papoutsi C (2018). Studying complexity in health services research: desperately seeking an overdue paradigm shift. BMC Med..

[35] Anderson R, Crabtree B, Steele D, McDaniel R (2005). Case study research: the view from complexity science. Qual Health Res.

[36] Greenhalgh T, Robert G, Bate P, Kyriakidou O, Macfarlane F, Peacock R (2004). How to spread good ideas: a systematic review of the literature on diffusion, dissemination, and sustainability of innovations in health service delivery and organization.

[37] Stirman SW, Gutner CA, Langdon K, Graham JR (2016). Bridging the gap between research and practice in mental health service settings: an overview of developments in implementation theory and research. Behav Ther.

[38] Thomas G (2011). A typology for the case study in social science following a review of definition, discourse, and structure. Qual Inquiry..

[39] Yin RK (2009). Case study research: design and methods.

[40] Davidoff F (2019). Understanding contexts: how explanatory theories can help. Implement Sci..

[41] Cargo M, Mercer SL (2008). The value and challenges of participatory research: strengthening its practice. Annu Rev Public Health.

[42] Kemmis S, McTaggart R, Denzin NK, Lincoln YS (2005). Participatory action research: communicative action and the public sphere. The SAGE handbook of qualitative research.

[43] Simons H. Case Study Research in Practice. London: SAGE Publications, Ltd; 2009. http://methods.sagepub.com/book/case-study-research-in-practice.

[44] Israel BA, Krieger J, Vlahov D, Ciske S, Foley M, Fortin P (2006). Challenges and facilitating factors in sustaining community-based participatory research partnerships: lessons learned from the Detroit, New York city and Seattle urban research centers. J Urban Health..

[45] Minkler M, Vasquez VB, Warner JR, Steussey H, Facente S (2006). Sowing the seeds for sustainable change: a community-based participatory research partnership for health promotion in Indiana, USA and its aftermath. Health Promot Int..

[46] Eisenhardt KM, Miles M, Huberman A (2002). Building theories from case study research. The Qualitative Researcher’s Companion.

[47] Goodyear MJ, Maybery DJ, Reupert AE, Morgan B, Cuff R, Carter H, et al. Best practice, next practice. Developing and implementing a parenting recovery intervention in adult mental health and family welfare services. In: Transgenerational Mental Health: the 5th International Conference on Families and Children with Parental Mental Health Challenges; 17-19 August; Basel, Switzerland. 2016.

[48] Allchin B, Weimand BM, O’Hanlon B, Goodyear M (2020). Continued capacity: Factors of importance for organizations to support continued Let’s Talk practice – a mixed-methods study. Int J Mental Health Nurs.

[49] Stirman SW, Kimberly J, Cook N, Calloway A, Castro F, Charns M (2012). The sustainability of new programs and innovations: a review of the empirical literature and recommendations for future research. Implement Sci..

[50] Westerlund A (2018). The role of implementation science in healthcare improvement efforts: investigating three complex interventions [Doctoral thesis, comprehensive summary].

[51] Westerlund A, Nilsen P, Sundberg L (2019). Implementation of implementation science knowledge: the research-practice gap paradox. Worldviews Evid Based Nurs..

[52] Foster KN, Maybery DJ, Reupert AE, Gladstone B, Grant A, Ruud T (2016). Family-focused practice in mental health care: an integrative review. Child Youth Serv..

[53] Lauritzen C, Reedtz C (2015). Knowledge transfer in the field of parental mental illness: Objectives, effective strategies, indicators of success, and sustainability. Int J Ment Health Syst..

[54] Maybery DJ, Reupert AE (2009). Parental mental illness: a review of barriers and issues for working with families and children. J Psychiatr Ment Health Nurs.

[55] Maybery DJ, Reupert AE (2006). Workforce capacity to respond to children whose parents have a mental illness. Aust N Z J Psychiatry.

[56] Stake RE (1995). The art of case study research.

[57] Eisenhardt KM, Graebner ME (2007). Theory building from cases: opportunities and challenges. Acad Manage J..

[58] Damschroder LJ, Aron DC, Keith RE, Kirsh SR, Alexander JA, Lowery JC (2009). Fostering implementation of health services research findings into practice: a consolidated framework for advancing implementation science. Implement Sci..

[59] Blase K, Fixsen D (2013). Core intervention components: Identifying and operationalizing what makes programs work ASPE Research Brief.

[60] Fixsen D, Blase K, Naoom S, Duda M. Implementation drivers: Assessing best practices. http://implementation.fpg.unc.edu/resources/implementation-drivers-assessing-best-practices?o=nirn: This document is based on the work of the National Implementation Research Network (NIRN). 2015. http://implementation.fpg.unc.edu/resources/implementation-drivers-assessing-best-practices?o=nirn.

[61] Blase K, Dyke M, Fixsen DL, Bailey FW, Kelly B, Perkins DF (2012). Implementation science: key concepts, themes and evidence for practitioners in educational psychology. Handbook of implementation science for psychology in education.

[62] Fixsen DL, Naoom SF, Blase KA, Friedman RM, Wallace F (2005). Implementation research: a synthesis of the literature.

[63] Wolcott H (1994). Transforming qualitative data.

[64] Fisher C, White N. River of Life Resource of knowledge sharing tools and methods. 2018. http://www.kstoolkit.org/river_of_life.

[65] Halcomb EJ, Davidson PM (2006). Is verbatim transcription of interview data always necessary?. Appl Nurs Res.

[66] Bryman A (2016). Social research methods.

[67] Lennox L, Doyle C, Reed JE, Bell D (2017). What makes a sustainability tool valuable, practical and useful in real-world healthcare practice? A mixed-methods study on the development of the Long Term Success Tool in Northwest London. BMJ Open..

[68] Aarons GA, Ehrhart MG, Farahnak LR, Sklar M (2014). Aligning leadership across systems and organizations to develop a strategic climate for evidence-based practice implementation. Annu Rev Public Health..

[69] Moullin JC, Ehrhart MG, Aarons GA (2018). The role of leadership in organizational implementation and sustainment in service agencies. Res Soc Work Pract..

[70] Aarons GA, Green AE, Trott E, Willging CE, Torres EM, Ehrhart MG (2016). The roles of system and organizational leadership in system-wide evidence-based intervention sustainment: a mixed-method study. Admin Policy Ment Health Ment Health Serv Res.

[71] Kerrissey M, Satterstrom P, Leydon N, Schiff G, Singer S (2017). Integrating: a managerial practice that enables implementation in fragmented health care environments. Health Care Manag Rev July Sept..

[72] Rouleau L (2005). Micro-practices of strategic sensemaking and sensegiving: how middle managers interpret and sell change every day*. J Manage Stud.

[73] Heyden MLM, Fourné SPL, Koene BAS, Werkman R, Ansari S (2017). Rethinking ‘top-down’ and ‘bottom-up’ roles of top and middle managers in organizational change: implications for employee support. J Manage Stud.

[74] Aarons GA, Sommerfeld DH, Willging CE (2011). The soft underbelly of system change: the role of leadership and organizational climate in turnover during statewide behavioral health reform. Psychol Serv..

[75] Fixsen DL, Blase KA, Naoom SF, Wallace F (2009). Core implementation components. Res Soc Work Pract..

[76] Neal JW, Neal ZP (2019). Implementation capital: merging frameworks of implementation outcomes and social capital to support the use of evidence-based practices. Implement Sci..

[77] Nicholson J, Valentine A (2019). Key informants specify core elements of peer supports for parents with serious mental illness. Front Psychiatry..

[78] Roper C, Grey F, Cadogan E. Co-production: Putting principles into practice in mental health contexts. Melbourne: Melbourne University; 2018. https://recoverylibrary.unimelb.edu.au/__data/assets/pdf_file/0010/2659969/Coproduction_putting-principles-into-practice.pdf.

[79] Elwyn G, Nelson E, Hager A, Price A (2019). Coproduction: when users define quality. BMJ Quality Saf.

[80] Batalden M, Batalden P, Margolis P, Seid M, Armstrong G, Opipari-Arrigan L (2016). Coproduction of healthcare service. BMJ Q Saf.

[81] Greenhalgh T, Robert G, Macfarlane F, Bate P, Kyriakidou O (2004). Diffusion of innovations in service organizations: systematic review and recommendations. Milbank Q.

[82] Clossey L, Mehnert K, Silva S (2011). Using appreciative inquiry to facilitate implementation of the recovery model in mental health agencies. Health Soc Work.

