# Supplementary material for: An explanatory model of factors enabling sustainability of let’s talk in an adult mental health service: a participatory case study
Source: Int J Ment Health Syst. 2020 Jul 9;14:48. doi: 10.1186/s13033-020-00380-9 (PMC7346490; doi:10.1186/s13033-020-00380-9)
Supplement: Supplementary file 1 — Additional file 1. Case Study participantion details (1.1) and session outline (1.2). [file 13033_2020_380_MOESM1_ESM.docx]

Additional file 1.1: Local Research Group Participants & Attendance

| **Participant** | **Gender** | **Meeting 1** | **Meeting 2** | **Meeting 3** | **Meeting 4** | **Meeting 5** | **Notes** |
| --- | --- | --- | --- | --- | --- | --- | --- |
| **Senior Manager** | M | **🗸** | **🗸** | **🗸** | **🗸** | **🗸** |  |
| **Quality & Safety** | F | **🗸** | **🗸** | **🗸** | **🗸** | **🗸** |  |
| **Parent** | F | Unable to participate | **🗸** | Unable to participate | Unable to participate | **🗸** | Met with researcher before meeting 2 to catch up & by phone after meeting 4 |
| **Manager** | F | Unable to participate | **🗸** | **🗸** | Annual leave | Unable to leave operational work | Clarified and fine-tuned influencer definitions and prioritised key influencers via email after meeting 4 and 5 |
| **FaPMI** | F | **🗸** | **🗸** | **🗸** | **🗸** | **🗸** |  |
| **Clinician F** | F | **🗸** | Annual leave | **🗸** | Not rostered on | Unable to leave clinical work | Clarified, fine-tuned influencer definitions and prioritised key influencers via email after meeting 4 and 5 |
| **Clinician S** | F |  |  |  |  |  | Not able to be released from duties |
| **Clinician J** | F |  |  |  |  |  | Not able to be released from duties |
| **Researcher** | F | **🗸** | **🗸** | **🗸** | **🗸** | **🗸** |  |
| **Total** |  | **5** | **6** | **6** | **4** | **5** |  |

Additional file 1.2: Case Study Session Outline

| **Session No.** | **Session objective** | **Tasks** | **Activities** | **Measure of aim met** | **Measure of participation process** |
| --- | --- | --- | --- | --- | --- |
| Pre-session email | Engage local research team in aim | Establish relationship with researcher | Overview of the study and where the case study fits |  |  |
|  |  | Establish an understanding of participants role | TOR | TOR signed |  |
| 1 (2 hrs) | Develop a shared understanding of implementation process | Establish a cohesive research team (20 min) | Introductions - name, role at Service, Engagement in LT at Service  2 truths and a lie  Develop group rules  Identify dual-purpose - research and service | Members participating | End of session feedback from participants via online qualtrics survey |
|  |  | Establish a shared understanding of the aim of case study (10 min) | Answer Q about the overview of the study, and where the case study fits |  | Reflective journal for the researcher |
|  |  | Establish a shared understanding of implementation journey identifying influences on practice and capacity (40 min) | *River of life tool* (Fisher & White, 2018; United Nations Institute for Training and Research (UNITAR), 2013)  Together identify a time frame for the journey  Each to identify points of local influence of practice and capacity as seen from their perspective within the river(rocks/ whirlpools)  Identify external influences outside the river  Raise questions that need more information for | Completed Implementation journey | Video session  Audio recording |
|  |  | Establish what other information is important/ add value that sits outside the group and the phase 2 data. (10 min) | Present Service’s data on practice and organisational capacity  Raise questions that need to collect more information for | List of other data to collect |  |
| 2  (1.5 hrs) | Develop a shared understanding of influencers of continued practice and capacity | Re-establish team cohesion | Warm-up by FaPMI coord |  |  |
|  |  | Review collected data   - practitioner use - implementation journey - implementation documents | Review practitioner data - phase 2 and current data.  Review assumptions and ideas raised in implementation journey activity  Create space for further questions to be raised | More questions raised | Analysis of sticky notes  Framelaps and audio  End of session feedback form |
|  |  | Introduce the generic framework for sustainability used for coding | Gave a rationale for using general framework for sustainability for coding data from implementation journey activity and presented developing theme matrix |  |  |
|  |  | Identify influencers of continued practice | Each brainstorm with own sticky notes (initial at bottom). Different colour for practice and capacity  Place on the wall so can be seen by all  Cluster influencers into categories using group consensus | Matrix of influencers |  |
|  |  | Identify influencers of continued capacity |  |  |  |
| 3  (1.5 hrs) | Compare key influencers to literature | Re-establish team cohesion | Warm-up | Refined theme matrix with descriptions matching data | Framelaps and audio  Work done in each pair  End of session feedback form |
|  |  | Present established frameworks that have shaped the theme matrix | Present the CFIR, active implementation and refresh re general framework for sustainability. |  |  |
|  |  | Review and refine developing themes matrix | Pairs reviewed a section of themes, their description and data checking   - themes reflected the data - if the themes picked up on what they had wanted to convey about key influencers. - Decide if theme should be kept, rolled into another or removed |  |  |
| 4  (2 hrs) | Prioritise key influencers | Re-establish team cohesion | Warm-up | Summation matrix | Priorities from each team member  End of session feedback form |
|  |  | Review the explanatory model | Present model of themes based on the generic framework for sustainability for discussion of its fit to the organisation |  |  |
|  |  | Review influencers matrix | Review and refine themes and description matrix of influencers of both practitioner use and organisational capacity |  |  |
|  |  | Identify key influencers | Each have 5 dots to allocate on the matrix as ones they see as 5 most important for practitioner use and 5 most important for organisations capacity |  |  |
| 5  (1.5 hrs) | Refine the explanatory model and plan future | Re-establish team cohesion (15 min) | Warm-up - 3 questions  Share 1 question at a time   - Something important did this morning - Some question came to the group with today - For this process not to feel unfinished what do you think we need to do today   Follow up from last time - what about LT that enabled sustaining |  |  |
|  |  | Review model of key influencers (10 min) | Review of key influencers model  Similarities/ Surprises/ differences & why |  |  |
|  |  | Explore purpose for model/ the So What (35 min) | So What- individually consider   - What could be the impact of the explanatory model from their standpoint - what is its purpose - What is the most important learning from the influencer matrix and explanatory model   Share as a group | Purpose list | Purpose from each member |
|  |  | Establish an action plan - what next (20 min) | Given these purposes and potential impact, what actions does who have to take  Brainstorm into action plan identifying actors, audience and timeframe | Action plan | Feedback form: |
